# Genotypes of *Acropora cervicornis* in Florida show resistance to either elevated nutrients or disease, but not both in combination

**DOI:** 10.1371/journal.pone.0320378

**Published:** 2025-03-26

**Authors:** Ana M. Palacio-Castro, Danielle Kroesche, Ian C. Enochs, Chris Kelble, Ian Smith, Andrew C. Baker, Stephanie M. Rosales

**Affiliations:** 1 Cooperative Institute for Marine and Atmospheric Studies, University of Miami, Miami, Florida, United States of America; 2 Atlantic Oceanographic and Meteorological Laboratory, NOAA, Miami, Florida, United States of America; 3 Department of Marine Biology and Ecology, University of Miami, Miami, Florida, United States of America; 4 NOVA Southeastern University, Davie, Florida, United States of America; King Abdulaziz University, SAUDI ARABIA

## Abstract

Coral restoration programs are expanding to revive coral populations and ecosystem services, but local and global stressors still threaten coral survival. In the Caribbean, the ESA-listed staghorn coral *Acropora cervicornis* has experienced profound declines due to multiple stressors, including disease and nutrient pollution. We studied the impact of these two stressors on ten *A. cervicornis* genotypes for which disease susceptibility was previously ranked in a disease transmission experiment. Results showed that elevated ammonium, disease, and their combination negatively affected *A. cervicornis* survivorship, with variable susceptibility among genotypes. Three genotypes were susceptible to elevated ammonium alone and experienced mortality in up to 80% of their fragments. Exposure to a disease homogenate under ambient ammonium caused mortality in 100% of the fragments in four coral genotypes, intermediate mortality in five (33-66% of their fragments), and no mortality in one genotype. However, all genotypes experienced mortality (30-100% of their fragments) when exposed to both elevated ammonium and disease. Despite the detrimental effects of ammonium on coral survivorship, corals under elevated ammonium presented higher photochemical efficiency (*F*_*v*_*/F*_*m*_) of the algal symbionts. Disease susceptibility did not align with the genotypic ranking established in a previous study, suggesting that, while genotypes may vary in their disease resistance, rankings may change due to environmental factors or disease type. Regardless of individual susceptibility, our results suggest that water quality improvement is necessary for increasing *A. cervicornis* survivorship.

## Introduction

Coral diseases are responsible for significant declines in coral populations, particularly in the Caribbean [1–6], which comprises just 8% of the world’s coral reefs by area, but reports over 70% of all coral diseases [7]. A variety of taxa, including bacteria, fungi, viruses, and protozoa have been associated with coral diseases [8–11], but despite extensive efforts to identify the causative agents, pathogen identification for most coral diseases and syndromes remains elusive [12,13].

*Acropora cervicornis* and *A. palmata* are the only two fast-growing branching scleractinian corals in the Caribbean and western Atlantic region. These species perform unique ecological functions and disproportionately contribute to reef growth, coastline protection, and essential habitat formation [14,15]. Unfortunately, *Acropora* populations have been severely impacted by diseases throughout the Caribbean, with declines of up to 90% between the 1970s and 1990s attributed to white band disease (WBD) [2]. Furthermore, the remaining populations of *Acropora* spp. continue to experience mortality and tissue loss associated with diseases and syndromes [16] as well as heat-induced coral bleaching [17]. Previous research has suggested bacteria in the orders *Vibronales*, *Rickettsiales*, and *Flavobacteriales* as candidate causative agents for WBD [8,18–20], but a specific pathogen for WBD has not yet been identified.

In response to these population declines, *A. cervicornis* and *A. palmata* became the first coral species designated as threatened under the US Endangered Species Act (ESA) in 2006 [21]. Over the past few decades, numerous coral restoration programs have been established to recover *Acropora* spp. coral cover and restore their ecological function [16,22,23]. However, since reefs still face numerous anthropogenic and natural threats [24,25], restoration initiatives are now evaluating and adopting population enhancement practices, such as selecting stress-resistant corals to improve restoration efficacy and the long-term persistence of the restored populations [26].

Previous studies have documented differences in survivorship and stress tolerance among *A. cervicornis* genotypes [27–31], providing valuable insights into prioritizing the outplanting of resilient corals in environments exposed to specific stressors. However, we still lack a comprehensive characterization of genotype-specific traits, their heritability, and their plasticity under different environmental conditions or multiple stressors [27,32,33]. For instance, while differences in disease resistance have been identified among *A. cervicornis* genotypes [27,28,30], these traits could be compromised under additional stressors such as elevated temperature [27], or elevated nutrients.

In the Caribbean, elevated nutrient levels in coastal reefs are often driven by anthropogenic factors, such as agricultural runoff and inadequate sewage treatment [34–36]. In Southeast Florida and the Florida Keys particularly, dissolved inorganic nitrogen (DIN = NH_4_ + NO_3_ + NO_2_) concentrations typically remain below 0.6 μM but can rise to 2.5–7.5 μM during nutrient input events, often coinciding with the onset of the wet season [36,37]. Elevated nutrient levels have been linked to various negative impacts on coral reefs, including increased macroalgal cover [38] and enhanced reef bioerosion [39]. Furthermore, the disproportionate increase in DIN relative to phosphorus (elevated N:P ratios) has been linked to reduced coral resilience to additional stressors, such as temperature [40].

Nutrient pollution can also interact with a coral’s resistance to disease [41–43]. Increased disease progression and prevalence have been reported in other Caribbean corals such as *Siderastrea siderea* [41,42], *Orbicella annularis,* and *O. franksi* [43] when exposed to nutrient enrichment. However, the mechanisms by which nutrients increase disease susceptibility are not fully understood. Evidence suggests that higher disease susceptibility could result from the effects of elevated nutrients on corals’ symbiotic communities, rather than the direct effects of nutrients on the coral host [44,45]. For instance, higher densities of algal symbionts (Family Symbiodiniaceae), a common phenomenon in corals exposed to nutrient enrichment [46,47], can depress the expression of coral immune-related genes [44]. Elevated nutrients can also disrupt coral’s prokaryotic communities, leading to dysbiosis [45]. In *A. cervicornis,* elevated nutrients seem to marginally affect microbiome composition [29,48], but correlate with an increase in the relative abundance of Aquarickettsia [48], a bacterial genus associated with reduced disease resistance [49].

To assess the effects of elevated ammonium (NH_4_) on *A. cervicornis* response to disease, we used ten nursery-reared coral genotypes that were previously tested in a disease transmission experiment [28]. These genotypes presented a range of disease transmission risks from 0 to 1, offering a full range of disease susceptibility [28]. Coral survivorship, algal symbiont composition, and photochemical efficiency (*F*_*v*_*/F*_*m*_) of the genotypes were compared under a full factorial design, including preconditioning to ambient nutrients followed by exposure to a placebo slurry (Ambient+Placebo), preconditioning to ambient nutrients followed by exposure to a diseased slurry (Ambient+Disease), preconditioning to elevated NH_4_ followed by exposure to a placebo slurry (elevated NH_4_ + Placebo), and preconditioning to elevated NH_4_ followed by exposure to a diseased slurry (elevated NH_4_ + Disease). We aimed to determine if the addition of NH_4_ changed *A. cervicornis* disease susceptibility and if changes in disease susceptibility were associated with disturbance of the Symbiodiniaceae communities. We identified genotypes resistant to either nutrients or disease but not the combination of the two. However, the disease susceptibility ranking by genotype was different from that previously reported.

## Materials and methods

### Coral collection and maintenance

Coral fragments from 10 nursery-reared *Acropora cervicornis* genotypes (n = 240; 24 per genotype) were donated during the summer of 2020 by three South Florida coral nurseries. Coral fragments from the University of Miami’s Rescue a Reef nursery were collected offshore of Key Biscayne, Miami (25.68°N, 80.11°W); fragments from the Coral Restoration Foundation were collected offshore of Tavernier in the Upper Keys (24.99°N, 80.43°W); and fragments from the Florida Fish and Wildlife Conservation Commission were collected offshore of Marathon in the Middle Keys (S1 Fig, S1 Table). All fragments were 5-8 cm long single branches with one apical tip. From these genotypes, four were previously characterized as resistant to disease (Acerv2, Cooper-9, K2, and FM9; risk of disease transmission 0 - 0.3); one as intermediate (U44; disease transmission 0.39); and five as susceptible (Kelsey-1, Elkhorn, FM6, FM14 and FM19, disease transmission between 0.5 and 1; S1 Table) [28]. After collection at their respective nursery, the corals were transported to the University of Miami Cooperative Institute for Marine and Atmospheric Studies (CIMAS) and the NOAA Atlantic Oceanographic and Meteorological Laboratory (AOML) Experimental Reef Lab (ERL). The fragments were mounted on numbered acrylic tags and acclimated to the lab conditions in eight independent ~180 L flow-through tanks [50] for six weeks. During the acclimation period, all tanks were maintained at 28°C, consistent with the approximate temperature in the nurseries at the time of collection. The temperature in each tank was measured with a high-resolution resistive temperature detector (TTD25C, ProSense) and adjusted to the target levels with a 400 W submersible heater and a solenoid-operated titanium chiller coil [50]. Light was supplied by Hydra 64 HD LED Reef lights under a 12:12 light-dark schedule, which included 1 h “up and dawn” ramp and 10 h at a PAR peak of ~350 μmol m^−2^ s^−1^. Filtered (1 μm) and UV-treated seawater from Bear Cut in Virginia Key, Florida was supplied at a rate of 200 mL/min. The coral fragments were fed daily with 1.0 g of ReefRoids in separate feeding tanks and transferred back to the experimental tanks after 1 h.

### Nutrient treatments

**Fig 1:**
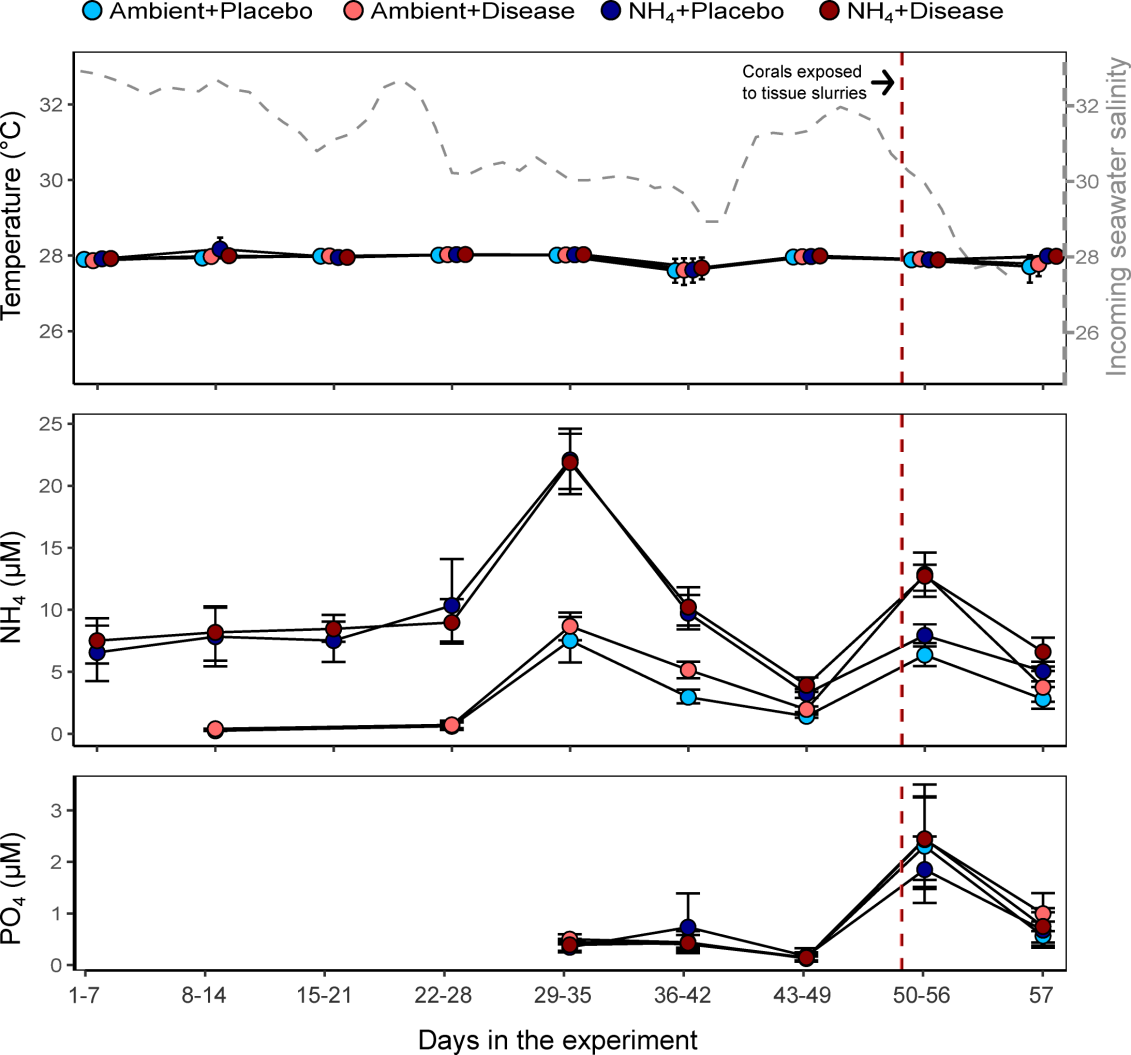
Measured experimental conditions. The colors represent the treatment combination assigned to each tank (nutrients + disease). However, disease was only applied on day 48. The gray line in the top panel represents the salinity of the incoming water from Biscayne Bay. The red vertical line shows the day when the tanks were dosed with tissue slurries. Values are shown as weekly averages for each treatment (7-day mean ± 95% CI).

After the acclimation period, all fragments of each genotype were evenly and haphazardly assigned to two nutrient treatments: ambient nutrients (Ambient) or elevated ammonium (NH_4_). Each nutrient treatment was replicated in four independent tanks (n = 3 fragments per genotype per tank) and maintained under the same temperature, water flow, and light conditions as the acclimation period. For ~1.5 months (47 d), Ambient tanks were maintained under nutrient levels consistent with the values in Virginia Key, FL, while elevated NH_4_ tanks were dosed with NH_4_Cl [3 mM] every 15 minutes using peristaltic pumps. The initial NH_4_ dose volume was 10 mL of the stock solution, targeting a ~10 μM increase in NH_4_ concentration. These values were calculated to account for the dilution of the nutrients resulting from adding new ambient seawater to the tanks (200 mL/min in a total tank volume of 180 L). However, after detecting higher than normal NH_4_ concentrations in the incoming seawater from Biscayne Bay, the NH_4_ dose volume was lowered to 5 mL of the stock solution, targeting ~5 μM NH_4_ increase above ambient values (Fig 1).

Water samples (~40 mL) were collected to monitor NH_4_ levels in the treatments and immediately refrigerated at 4^o^C. The elevated NH_4_ tanks were sampled daily, but the Ambient tanks were sampled less frequently (~2-3 days, and no samples were collected during weeks 1 and 3 of the experiment). Nutrient concentrations were measured at NOAA-AOML using an AA3 nutrient analyzer (Seal Analytical, Southampton, UK). The instrument was calibrated before each run using standard solutions and procedures [51]. Initially, only NH_4_ was monitored, but after high NH_4_ concentrations in the source seawater were detected, additional measurements of PO_4_ were included (Fig 1).

### Photochemical efficiency (*F*_*v*_ */F*_*m*_)

The photochemical efficiency of each fragment (*F*_*v*_*/F*_*m*_) was measured every week during the nutrient exposure period (days 1 to 47) to assess the effects of elevated NH_4_ on algal symbiont community function in *A. cervicornis* [52]. Corals were dark-adapted for 30 minutes and measured using a Maxi Imaging-PAM fluorometer (I-PAM, Walz, Effeltrich, Germany). Monitoring of *F*_*v*_*/F*_*m*_ stopped once the tissue slurries were applied to the experimental fragments to avoid cross-contamination between the placebo and disease treatments. The effect of nutrients on *F*_*v*_*/F*_*m*_ was analyzed with a linear mixed model that included nutrient treatment, genotype, and day of the experiment as fixed interacting effects. Coral fragment was included as a random effect to account for repeated measurements of the same fragments during different time points. The models were run with the lme4 1.1-17 package [53] for R 3.4.3 [54] and pairwise (Tukey’s HSD) comparisons for significant factors were performed with emmeans 1.1.3 [55] with an alpha value of 0.05.

### Symbiodiniaceae community

Three tissue samples per genotype and nutrient treatment were collected on day 47 from a subset of fragments (n=60). Small tissue biopsies (~4 polyps) were collected using a new razor blade per coral and were preserved in DNA/RNA Shield (Zymo Research). Total genomic DNA was extracted using the MagBead RNA/DNA extraction kit (Zymo Research). The tissue samples were first homogenized in the collection tubes using an MP bead beater for 30 s at 4 m/s and then centrifuged at 10,000g for 60 s. Proteinase K was added for 30 minutes. A total of 200µl of supernatant was used for extractions using the semi-automatic Kingfisher Flex (Thermofisher) and extracted samples were stored in a -80ºC freezer until they were used for quantitative PCR (qPCR) analysis.

The symbiont to host cell ratio (S/H) was used as a proxy to quantify differences in algal symbiont abundance relative to the coral host tissue as a response to the nutrient treatments [56]. S/H ratios were estimated using quantitative PCR (qPCR) assays that target the actin gene in the genus *Symbiodinium*, *Breviolum*, *Cladocopium,* or *Durusdinium* [57,58] and the Calmodulin (CAM) gene in *Acropora* [59]. S/H ratios were calculated using the r package SteponeR [60]. This R package uses the formula S/H cell ratio = 2^Ct(host)−Ct(symbiont)^, divided by the symbiont to host ploidy ratio (1/2), DNA extraction efficiency ratio (0.828), and target locus copy number ratio (9/1) [56–58]. To quantify the differences in S/H cell ratio by genotype and nutrient treatment the ratios were analyzed with a linear mixed model that included nutrient treatment and genotype as fixed interactive effects, and tank as a random effect. The models were run with the lme4 1.1-17 package. Non-significant factors were dropped with the function ’step’ and pairwise (Tukey’s HSD) comparisons for significant factors were performed with emmeans 1.1.3 with an alpha value of 0.05.

### Disease treatments

After 48 days of exposure to the nutrient treatment, half of each nutrient cohort was exposed to a diseased coral tissue slurry (Disease) and half to a healthy coral tissue slurry (Placebo). This resulted in a multi-factorial experiment with four treatments of combined stressors replicated in two independent tanks: ambient nutrients and placebo (Ambient+Placebo), ambient nutrients and disease (Ambient+Disease), elevated nutrients and placebo (elevated NH_4_+Placebo), and elevated nutrients and disease (elevated NH_4_+Disease).

Tissue slurries were made following guidance from a previous disease exposure study [27]. Briefly, fragments of *A. cervicornis* characterized as “visually healthy” and fragments with lesions indicative of WBD were collected from the University of Miami - Rescue a Reef nursery. The healthy and diseased corals were obtained from two different locations and maintained in separate tanks to confirm the absence of disease signs in the placebo (visually healthy) fragments and active lesion progression in disease fragments (for 3 and 2 days, respectively). The slurries were made using six healthy and seven diseased fragments which were blasted using an airbrush with 0.22 μm filtered and autoclaved seawater. Each slurry was transferred to a Falcon tube containing sterile steel beads, and vortexed for 10 minutes to homogenize the mixture and break up mucus [61].

To facilitate infection, all experimental corals were scraped around their circumference near their base using a new razor blade for each fragment before exposing them to the slurries [27]. All fragments from a tank were then transferred to a closed 21 L glass aquarium containing 14 L of seawater from their respective nutrient treatment. Depending on the treatment, the aquaria were dosed with either 31 mL of placebo or disease slurry. All corals were maintained in the closed aquaria with recirculating pumps for ~ 12 h to facilitate exposure to the pathogen. After this, the corals, along with the water from the 21 L glass aquaria, were returned to their original flowthrough tanks and nutrient treatments, where the appearance of disease lesions was monitored for nine days (until day 57).

### Tissue loss and survivorship probabilities

The appearance of lesions or tissue loss was monitored daily to determine the effects of single and combined stressors on the genotypes’ survivorship. Mortality was recorded once each coral exhibited tissue loss since the whole fragment usually died within a day of the first appearance of a lesion. Consequently, percentage mortality represents the proportion of fragments that developed lesions and not the percentage of tissue affected in each coral. Monitoring of the fragments was stopped nine days after exposure to the slurries because tissue loss had slowed on day eight and none of the fragments experienced mortality on day nine. Survival probabilities were calculated for each genotype and treatment combo (nutrients x slurry) using the Kaplan-Meier estimate [62] with the R packages survival 2.38 [63] and survminer 0.4.6 [64]. Log-rank tests were used to compare the survival curves for each genotype, treatment, and combined genotype and treatment. All data and code for the statistical analysis are available on Zenodo [65].

## Results

### Treatment conditions

Temperature was maintained at ~28 °C in all four treatments (Ambient+Placebo = 27.89 ± 0.34 *sd*, Ambient+Disease = 27.79 ± 0.99, NH_4_+Placebo = 27.99 ± 0.33, and NH_4_+Disease = 27.94 ± 0.24; Fig 1). During the first four weeks of nutrient treatments, average NH_4_ concentration was 0.51 μM (± 0.21 SD) in the ambient tanks, and 8.17 μM (± 1.13) in the NH_4_ tanks. However, in week 5, nutrient concentrations in the incoming seawater spiked to 8.10 μM NH_4_ (± 0.82) in ambient nutrients, and 21.97 μM (± 0.14) in elevated NH_4_. This spike (in October 2020) coincided with a period of heavy rain and a mass fish kill in Biscayne Bay [66] and a reduction in the salinity of the incoming water (Fig 1 top panel). After detecting this nutrient spike, nutrient dosing was reduced by half to avoid excessively high nutrient levels in the NH_4_ treatments. During weeks 6 and 7, NH_4_ concentration in the incoming water started to decline and by week 6 it was 4.06 μM (± 1.56) in the ambient tanks and 9.98 μM (±0.35) in the elevated NH_4_. By week 7 it had declined to 1.70 μM (± 0.40) in the ambient tanks and 3.59 (± 0.47) in elevated NH_4_ (Fig 1). In week 8, the addition of tissue slurries temporarily increased NH_4_ and PO_4_, with NH_4_ concentrations reaching 7.15 μM (± 1.12) in the tanks dosed with placebo and 12.79 μM (± 0.11) in the tanks dosed with disease slurry before declining again in week 9 (Fig 1)

### Photochemical efficiency *(Fv/Fm)*

**Fig 2:**
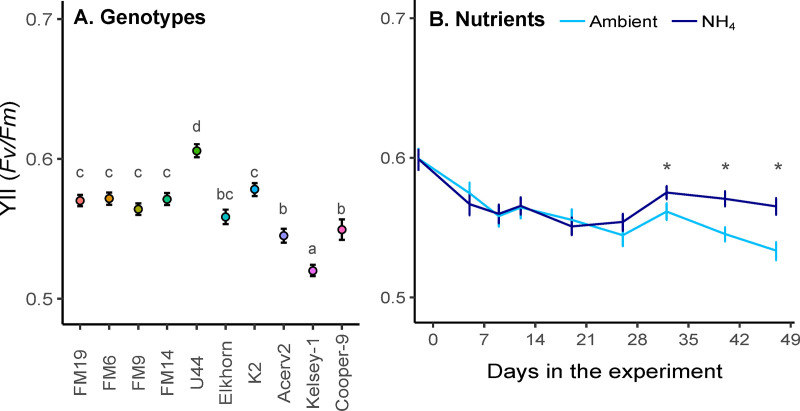
Maximum quantum yield (*F*_*v*_*/F*_*m*_; mean ± 95% CI) of *A. cervicornis*. (A) *F*_*v*_*/F*_*m*_ across time points for the ten genotypes used in the experiment. The letters represent the TukeyHSD groups (i.e., *F*_*v*_*/F*_*m*_ in genotypes with shared letters are not significantly different). Genotypes are arranged by descending survivorship probabilities on the final day of the experiment from left (highest overall survivorship across treatments) to right (lowest overall survivorship). (B) *F*_*v*_*/F*_*m*_ by nutrient treatment and time points before adding the tissue slurries. The asterisks denote the time points when significant differences occur between the nutrient treatments.

Nutrient treatment (p<0.001, F val=9.07), genotype (p<0.001, F val=30.08), and day of the experiment (p<0.001, F val=90.74) had significant effects on the *F*_*v*_*/F*_*m*_ (S3 Table). However, these differences were generally small with all corals presenting *F*_*v*_*/F*_*m*_ values above 0.5. Across nutrient treatments and days, U44 had the highest *F*_*v*_*/F*_*m*_ compared to the rest of the genotypes (0.606 + 0.004, p<0.05), and Kelsey-1 had the lowest (0.520 + 0.004; Fig 2A). Corals exposed to elevated NH_4_ presented higher *F*_*v*_*/F*_*m*_ values compared to corals in Ambient nutrients, but these differences were not significant during the early days of the experiment (days 1-20, Fig 2B). There was no interaction between genotype and nutrient treatment, with all genotypes exhibiting similar responses to the nutrient treatments over time.

### Symbiodiniaceae community

**Fig 3:**
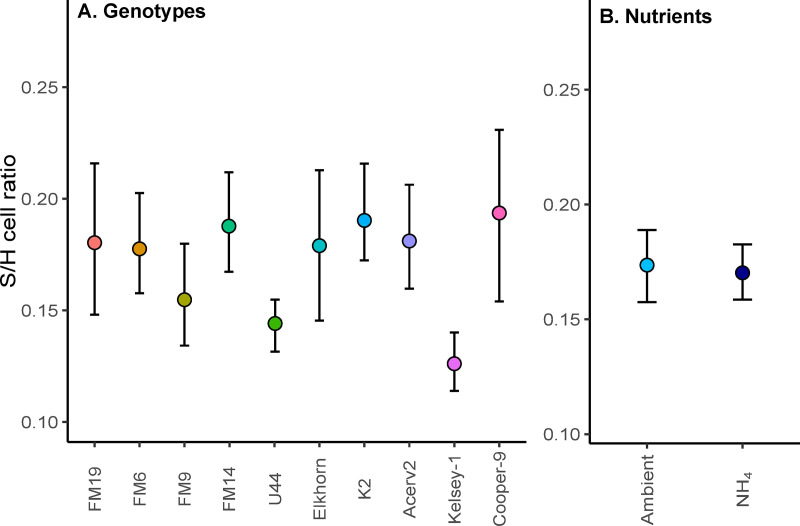
Symbiont to host cell ratio (S/H; mean± 95% CI) of *A. cervicornis.* (A) S/H on day 47 for all the different genotypes used in the experiment. Genotypes are arranged by descending survivorship probabilities on the final day of the experiment from left (highest overall survivorship across treatments) to right (lowest overall survivorship). (B) S/H on day 47 by nutrient treatment (all genotypes combined).

All *A. cervicornis* genotypes hosted algal symbionts in the genus *Symbiodinium* (likely *S. fittii*) with no other genera being detected by qPCR. There were no statistical differences among the S/H cell ratios in the corals under elevated NH_4_ compared to Ambient nutrients (p=0.86, F val=0.03; Fig 3; S5 Table). Coral genotype showed a significant effect on S/H model (p=0.03, F val=2.43), but post-hoc Tukey comparisons were not significant among the genotypes (p>0.05; S6 Table). The interaction between nutrient treatment and genotype on S/H was also not significant (p=0.66, F val=0.75).

### Coral survivorship

**Fig 4:**
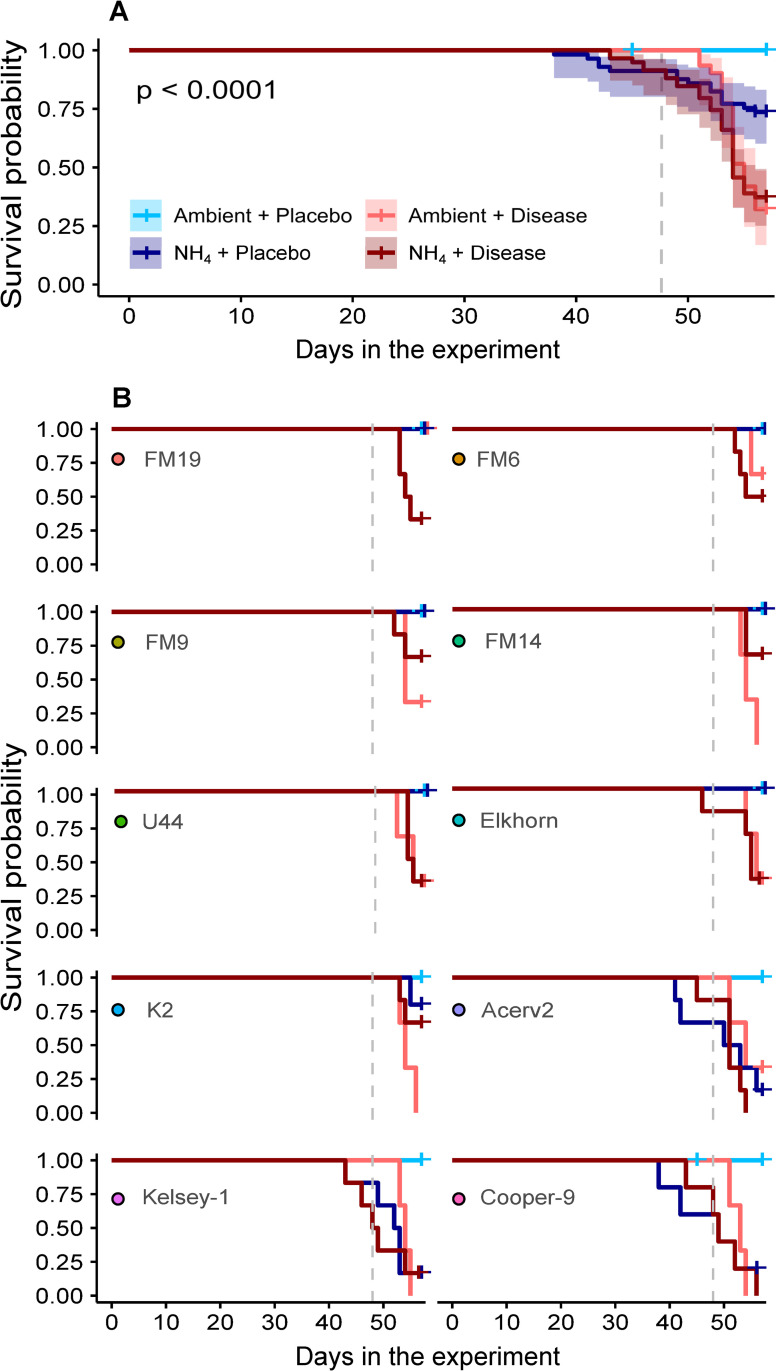
Survival probabilities in *A. cervicornis* exposed to Ambient or elevated NH_4_ in combination with exposure to a placebo or disease slurry. (A) Overall survival probabilities by treatment (± 95% CI). (B) Survival probabilities by treatment and genotype. The vertical dashed lines represent the day when the corals were exposed to the tissue slurries.

There was no mortality across genotypes in the Ambient+Placebo treatment (Fig 4A). On average, exposure to NH_4_+Placebo reduced survivorship probabilities to 83% (log-rank *p<*0.05), but this reduction in survivorship was observed in only four genotypes (Fig 4B). From these, three genotypes started experiencing tissue loss before exposure to the placebo slurry (Cooper, day 38; Acerv2, day 41; Kelsey-1, day 43), and their survivorship probabilities dropped below 20% by the end of the experiment (day 56). The remaining nutrient-susceptible genotype (K2) experienced tissue loss after placebo exposure (day 55), and had a higher survivorship probability by the end of the experiment (80%), compared to the other three NH_4_ susceptible genotypes (Fig 4B).

All but one genotype (FM19) experienced tissue loss in the Ambient+Disease treatment after exposure to the disease slurry. By the end of the experiment, survivorship probabilities were reduced to 0% for Cooper-9, Kelsey-1, K-2, and FM14; 33% for Acerv2, Elkhorn, U44, and FM9; and 66% for FM6 (Fig 4B).

All genotypes, including FM19, experienced tissue loss in the NH_4_+Disease treatment either before or after exposure to the disease slurry. However, overall mortality (all genotypes pooled) was not significantly different between the NH_4_+Disease and Ambient+Disease (Fig 4A). Genotypes Cooper, Acerv2, Kelsey-1, and K2 in the NH_4_+Disease treatment started experiencing mortality before exposure to the disease slurry, in agreement with their susceptibility to elevated NH_4_. The rest of the genotypes only experienced mortality after the addition of the slurry. Survivorship probabilities were reduced to 0% in Cooper-9 and Acerv2, to 16% in Kelsey-1, 33% in Elkhorn, U44 and FM19, 50% in FM6, and 67% in FM14, FM9, and K2 (Fig 4B; Table 1).

**Table 1:** Final survival probabilities of *A. cervicornis* genotypes.

Genotype	Treatment	Survivorship	Standard Error	Survivorship in Miller et al. [28]
FM19	Ambient+Disease	1	NA	0.5
	NH4+Placebo	1	NA	
	NH4+Disease	0.33	0.19	
FM6	Ambient+Disease	0.67	0.27	0.35
	NH4+Placebo	1	NA	
	NH4+Disease	0.50	0.20	
FM9	Ambient+Disease	0.33	0.27	1
	NH4+Placebo	1	NA	
	NH4+Disease	0.67	0.19	
FM14	Ambient+Disease	0.00	NA	0.35
	NH4+Placebo	1	NA	
	NH4+Disease	0.67	0.19	
U44	Ambient+Disease	0.33	0.27	0.61
	NH4+Placebo	1	NA	
	NH4+Disease	0.33	0.19	
Elkhorn	Ambient+Disease	0.33	0.27	0
	NH4+Placebo	1	NA	
	NH4+Disease	0.33	0.19	
K2	Ambient+Disease	0.00	NA	0.78
	NH4+Placebo	0.80	0.18	
	NH4+Disease	0.67	0.19	
Acerv2	Ambient+Disease	0.33	0.27	0.7
	NH4+Placebo	0.17	0.15	
	NH4+Disease	0.00	NA	
Kelsey-1	Ambient+Disease	0.00	NA	0.1
	NH4+Placebo	0.17	0.15	
	NH4+Disease	0.17	0.15	
Cooper-9	Ambient+Disease	0.00	NA	0.7
	NH4+Placebo	0.20	0.18	
	NH4+Disease	0.00	NA	

The Survivorship and Standard Error columns represent the survival probabilities at the end of the experiment (day 57) for corals exposed to Ambient+Disease, NH₄+Placebo, and NH₄+Disease treatments. The Survivorship in Miller et al. [28] column represents 1 minus the risk of transmission reported in Miller et al. [28].

## Discussion

Identifying coral genotypes resistant to multiple stressors is key for improving restoration success since this can help select genotypes to be outplanted in zones exposed to specific threats [26,67]. Here, we examined the survivorship of ten *A. cervicornis* genotypes previously ranked for disease resistance. These genotypes were evaluated under a disease challenge alone, elevated NH_4_ alone, and the combination of these two stressors.

### Disease response under ambient nutrients

We found significant differences in disease susceptibility among *A. cervicornis* genotypes after being exposed to ambient nutrients and a disease slurry. Only one genotype was completely resistant to disease (FM19), five genotypes exhibited variable survivorship (33% to 66% of the fragments), while four genotypes had no survivorship (Fig 2). Based on our results, genotype FM19 may be promising to outplant in areas with above average disease outbreaks. However, for this approach to succeed, it is crucial to verify that the observed resistance of a coral genotype persists over time and across varying environmental conditions (i.e., if it is a fixed effect deriving from, for example, the coral’s genetic makeup or its stable association with specific symbiotic communities). In this study, we did not identify differences in the Symbiodiniaceae community that can be associated with disease resistance in the coral genotypes (Fig 3A), but it is possible that more fine differences in the algal community (e.g., to the species or strain level), the prokaryotic community [49], or the coral genetic make-up of the corals [68] could still play a role on *A. cervicornis* survivorship when exposed to disease. Further exploration of the holobiont composition and genetic traits of FM19 can help elucidate the mechanisms of its disease resistance and provide insights into the longevity of its resilience under varying environmental conditions.

To incorporate genotypic information into restoration, it is also essential to ensure that resistance to one stressor does not result in unintended trade-offs that can be detrimental to the long-term sustainability of the coral population (e.g., higher susceptibility to other stressors, lower reproductive output, or reduced growth rates) [69,70]. Here, all coral genotypes exhibited *F*_*v*_*/F*_*m*_ values characteristic of healthy algal symbiont communities, but other performance metrics besides survivorship were not measured.

Among the corals exposed to ambient nutrients and disease, genotype FM19 had the lowest disease transmission rate in this experiment (transmission rate = 0). However, FM19 resistance may not persist across multiple disease challenges since this genotype was previously characterized as disease-susceptible by Miller et al. [28] (transmission rate = 0.5). Similarly, FM9, Acer 2, Cooper, and K2 were previously among the most disease-resistant genotypes (transmission rate = 0-0.3), but they were among the susceptible genotypes in this study (Fig 4). This variability in resistance across experiments could suggest that some of the disease susceptibility traits in *A. cervicornis* might not be a result of fixed genetic effects but also modulated by the environmental history of the corals, including exposure to additional stressors or suboptimal conditions that can increase disease susceptibility [27,42]. Although in this study all coral fragments were common gardened in the laboratory for six weeks before initiating the experiment, the genotypes came from three different nurseries, one located near Miami, one in the Upper Keys and one in the Middle Keys (S1 Fig), and were therefore previously exposed to different environments [71]. To control for the impacts of environmental history, future studies should use genotypes that have been common-gardened in the same nursery prior to ex-situ experimentation, ensuring they experienced similar conditions in terms of temperature, nutrients, light, access to heterotrophic feeding, etc.

Additionally, it is possible that the pathogens involved in the infections differed between the two experiments; hence, the genotypes might exhibit different resistance levels to different diseases. In our experiment, all corals were exposed to the same disease slurries. In contrast, in Miller et al. [28], disease transmission occurred through direct contact with fragments exhibiting tissue loss. This last method could introduce additional variability in the disease transmission response, as different disease donors might not have the same virulence or could be affected by different pathogens. Indeed, a follow-up microbiome analysis of the Miller et al. [28] samples found that a putative pathogen was only found in 67% of diseased samples [72]. Future experiments could benefit from using isolated pathogens rather than disease homogenates or direct contact with diseased fragments [73]. Testing disease resistance with isolated microorganisms might reduce the variability associated with mixed-pathogen exposures and allow for a more precise evaluation of genotype-pathogen interactions.

### Elevated ammonium and *A. cervicornis* performance

The addition of ammonium (NH_4_) for over a month had a small and positive effect on the photochemical efficiency (*F*_*v*_*/F*_*m*_) of the algal symbionts, no effect on the Symbiodiniaceae abundance (relative to the host cells), but detrimental effects on coral survivorship (Figs 2B and 4), a pattern previously reported for *A. cervicornis* under elevated nutrients [59]. Differences in *F*_*v*_*/F*_*m*_ and survivorship were observed only after a month of nutrient addition, suggesting that chronic vs. acute nutrient enrichment might have differential effects on *A. cervicornis* performance. For example, significant disruption of the prokaryotic communities in *A. cervicornis* can take up to six weeks of nutrient exposure [74], and changes in growth rates might take up to a month [29,75]. This suggests that *A. cervicornis* might initially buffer the impacts of elevated nutrients, but this initial effect may be lost over the longer term [29,74,75].

Survivorship in the NH_4_+Placebo treatment was reduced in four of ten genotypes (Cooper, Acer2, Kelsey-1, and K2), with mortality observed in approximately 80% of their fragments. This result underscores the potential impact of sustained nutrient enrichment on *A. cervicornis* health, but also the variability of responses among genotypes. Corals are associated with diverse microorganisms that participate in nutrient cycling, and sustained nutrient enrichment in the NH_4_ treatments may alter the composition and abundance of members of the holobiont, impacting coral health [29,45]. Previous research also identified performance differences among *A. cervicornis* genotypes exposed to ammonium, with survivorship rates linked to two bacterial taxa (*Midichloriaceae* and *Spirochaetaceae*), suggesting that some coral microbiomes can be more resilient to nutrient stress [29].

During the nutrient spike, ammonium on the ambient treatments reached an average of ~ 8uM, highlighting the occurrence of high nutrient concentrations in Biscayne Bay that could impact the persistence and recovery of *A. cervicornis* in this area [76,77]. Despite the effects of elevated ammonium on coral survivorship and *F*_*v*_*/F*_*m*_, this treatment did not affect the abundance of the Symbiodiniaceae communities (Fig 3). However, these results should be interpreted with caution since our ambient tanks were exposed to a spike in NH_4_ a week before collecting the tissue samples, caused by high nutrient levels in the incoming seawater. Furthermore, at the time of tissue collections, ammonium was notably similar between ambient and elevated NH_4_ (Fig 1). Unlike the host and prokaryotic community, symbiont densities may respond quickly to nitrogen concentrations [78] and thus these results may be especially sensitive to time of sampling.

### Disease response under elevated ammonium

The overall mortality of corals exposed to disease under ambient and elevated NH_4_ was similar by the end of the experiment, reaching ~62-68% (Fig 4A). This appears to contrast with previous studies where nutrient enrichment has been found to exacerbate coral diseases [41–43]. However, we found important differences in the response to the combined stressors based on genotype (Fig 4). The only genotype with 100% survivorship under elevated NH_4_ alone and disease exposure alone (FM19) experienced mortality in ~ 67% of its fragments under the combined stressors. In contrast, pre-exposure to elevated NH_4_ did not exacerbate mortality in other genotypes, such as FM9, FM14, U44, or Elkhorn. Additionally, nutrient-sensitive genotypes, such as Acerv2, Kelsey-1, and Cooper-9 started experiencing mortality in the NH_4_+Disease treatment before exposure to the disease slurry, making it difficult to interpret if the subsequent mortality was the result of one or both stressors combined.

Another challenge to the interpretation of these results is that the Ambient+Disease corals were exposed to two periods of elevated nutrients. One before disease exposure, characterized by elevated NH_4_; and a second one after the application of the tissue slurries, characterized by elevated NH_4_ and PO_4_. Both periods of nutrient enrichment coincided with reduced salinity in the incoming seawater (Fig 1), suggesting that runoff contributed to the nutrient increases. Additionally, the second nutrient spike coincided with the application of the placebo and diseased tissue slurries. Although the coral fragments were initially exposed to the slurries in separate closed aquaria, they were later transferred back to their flowthrough systems along with the water from the closed exposure phase, potentially introducing additional nutrients into the tanks. Based on the flow rates of our systems (~200 mL/min), a full water exchange would occur within 13 hours. To better understand the interactions between elevated nutrients and disease, future studies may benefit from using artificial seawater to minimize natural nutrient variability common in facilities sourcing seawater from coastal environments. Additionally, when feasible, replacing tissue slurries with isolated bacterial cultures as a method for disease exposure [73] may also be preferable to prevent unwanted nutrient enrichment.

## Conclusions

Restoration efforts could benefit from identifying coral stocks with greater resistance to stressors such as disease and eutrophication to increase long-term coral survival, and thus restoration efficacy. Here, we found that some *A. cervicornis* genotypes were resistant to elevated NH_4_ or disease, but there were no genotypes resistant to both stressors combined. Additionally, our results did not align with previous genotypic rankings of disease susceptibility, suggesting that coral response may be impacted by previous environmental history, and/or that coral genotypes may vary in their susceptibility to different pathogens. Significant coral mortality under elevated ammonium alone, as well as the loss of disease resistance in one genotype exposed to combined nutrients and disease, indicate that improvement of water quality may be necessary to recover and sustain *A. cervicornis* populations.

## Supporting information

S1 Fig Geographical location of the coral nurseries. The gray shapes represent South Florida and the Florida Keys’ land. The color scale represents the ocean bathymetry obtained from the Second-generation Louvain la-Neuve Ice-ocean Model (SLIM, https://www.slim-ocean.be). The white dots demarcate the nurseries’ location. UM: University of Miami - Rescue a Reef, CRF: Coral Restoration Foundation, FWC: Florida Fish and Wildlife Conservation.
(DOCX)

S1 Table Genotypes used in the experiment and summary of their previous disease susceptibility based on Miller et al. (2019). (DOCX)

S2 Table Weekly conditions in the experimental treatments. (DOCX)

S3 Table *Fv/Fm* model. (DOCX)

S4 Table *Fv/Fm* pairwise comparisons among *A. cervicornis* genotypes. (DOCX)

S5 Table *Fv/Fm* Pairwise comparisons between the nutrient treatments. (DOCX)

S6 Table S/H cell ratio model. (DOCX)

S7 Table S/H cell ratio pairwise comparisons among *A. cervicornis* genotypes. (DOCX)
