# Supplementary material for: Genotypes of Acropora cervicornis in Florida show resistance to either elevated nutrients or disease, but not both in combination
Source: PLoS One. 2025 Mar 26;20(3):e0320378. doi: 10.1371/journal.pone.0320378 (PMC11940558; doi:10.1371/journal.pone.0320378)

**S1 Figure. Geographical location of the coral nurseries.** The gray shapes represent South Florida and the Florida Keys’ land. The color scale represents the ocean bathymetry. The white dots demarcate the nurseries’ location. UM: University of Miami - Rescue a Reef, CRF: Coral Restoration Foundation, FWC: Florida Fish and Wildlife Conservation. The map was generated in R using bathymetry data from the Second-generation Louvain la-Neuve Ice-ocean Model (SLIM, <https://www.slim-ocean.be>) and the nursery coordinates.


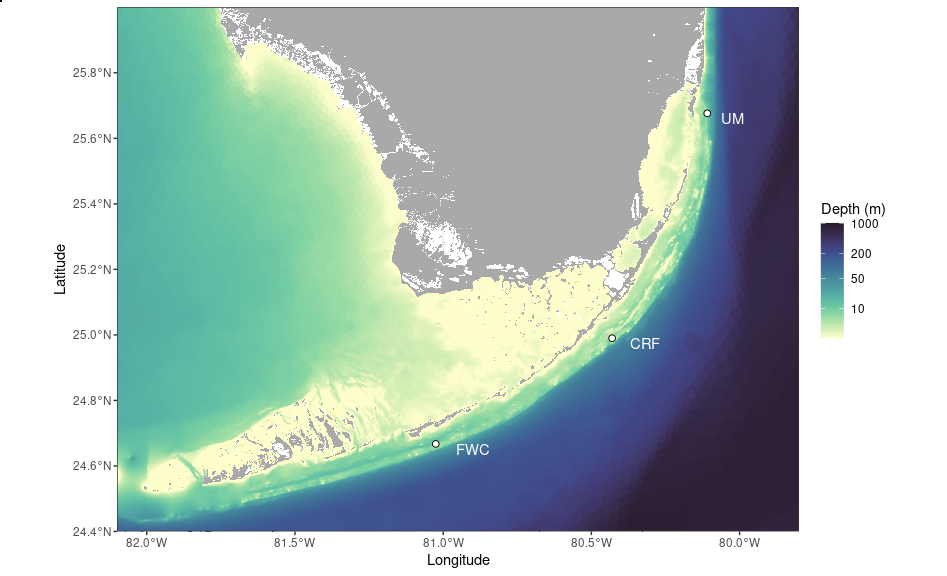

Supplement: S1 Fig — The gray shapes represent South Florida and the Florida Keys’ land. The color scale represents the ocean bathymetry obtained from the Second-generation Louvain la-Neuve Ice-ocean Model (SLIM, https://www.slim-ocean.be). The white dots demarcate the nurseries’ location. UM: University of Miami - Rescue a Reef, CRF: Coral Restoration Foundation, FWC: Florida Fish and Wildlife Conservation. (DOCX) [file pone.0320378.s001.docx]
