# Supplementary material for: Genotypes of Acropora cervicornis in Florida show resistance to either elevated nutrients or disease, but not both in combination
Source: PLoS One. 2025 Mar 26;20(3):e0320378. doi: 10.1371/journal.pone.0320378 (PMC11940558; doi:10.1371/journal.pone.0320378)
Supplement: S1 Table — (DOCX) [file pone.0320378.s002.docx]

**S1 Table. Experimental corals.** Genotypes used in the experiment and summary of their previous disease susceptibility based on Miller et al. (2019). UM: University of Miami, CRF: Coral Restoration Foundation, FWC: Florida Fish and Wildlife Conservation.

| **Donor Nursery** | **Nursery ID and this study** | **Miller et al.**  **(2019) ID** | **Disease Susceptibility**  **in Miller et al. (2019) -**  **(risk of transmission)** | **Algal symbionts detected**  **(this study)** |
| --- | --- | --- | --- | --- |
| UM | Acerv2 | C26 | Resistant - (0.3) | *Symbiodinium* |
| UM | Cooper-9 | C24 | Resistant - (0.3) | *Symbiodinium* |
| UM | Kelsey-1 | C17 | Susceptible - (0.9) | *Symbiodinium* |
| UM | Elkhorn | C21 | Susceptible - (1) | *Symbiodinium* |
| CRF | U44 | C3 | Intermediate - (0.39) | *Symbiodinium* |
| CRF | K2 | C6 | Resistant - (0.22) | *Symbiodinium* |
| FWC | FM6 | C8 | Susceptible - (0.65) | *Symbiodinium* |
| FWC | FM9 | C9 | Resistant - (0) | *Symbiodinium* |
| FWC | FM14 | C16 | Susceptible - (0.65) | *Symbiodinium* |
| FWC | FM19 | C13 | Susceptible - (0.5) | *Symbiodinium* |
