# Supplementary material for: Genotypes of Acropora cervicornis in Florida show resistance to either elevated nutrients or disease, but not both in combination
Source: PLoS One. 2025 Mar 26;20(3):e0320378. doi: 10.1371/journal.pone.0320378 (PMC11940558; doi:10.1371/journal.pone.0320378)
Supplement: S2 Table — (DOCX) [file pone.0320378.s003.docx]

**S2 Table. Weekly conditions in the experimental treatments.** Weekly mean ± (sd) for salinity, temperature (Temp.), ammonium (NH_4_) and phosphate (PO_4_)

|  | **Treatment** | **Week 1** | **Week 2** | **Week 3** | **Week 4** | **Week 5** | **Week 6** | **Week 7** | **Week 8** | **Week 9** |
| --- | --- | --- | --- | --- | --- | --- | --- | --- | --- | --- |
| **Salinity** | Incoming water | 30.87±(0.57) | 30.65±(0.71) | 30.06±(0.21) | 29.75±(0.13) | 28.88±(1.07) | 30.1±(0.69) | 26.81±(0.86) | 28.39±(0.14) | ±() |
| **Temp.**  **(°C)** | Ambient + Placebo | 27.9±(0.15) | 27.94±(0.17) | 27.98±(0.02) | 28.01±(0.03) | 28.01±(0.03) | 27.6±(0.66) | 27.96±(0.1) | 27.89±(0.21) | 27.71±(0.69) |
|  | Ambient + Disease | 26.63±(2.89) | 27.98±(0.09) | 27.99±(0.03) | 28.02±(0.01) | 28.02±(0.03) | 27.62±(0.66) | 27.97±(0.13) | 27.91±(0.2) | 27.77±(0.51) |
|  | NH4 + Placebo | 27.91±(0.14) | 28.17±(0.55) | 27.95±(0.13) | 28.02±(0.01) | 28.02±(0.02) | 27.62±(0.64) | 27.97±(0.09) | 27.89±(0.22) | 27.99±(0.03) |
|  | NH4 + Disease | 27.92±(0.11) | 28±(0.06) | 27.95±(0.14) | 28.03±(0.01) | 28.03±(0.01) | 27.67±(0.57) | 27.99±(0.06) | 27.88±(0.23) | 27.98±(0.04) |
| **NH_4_**  **(μM)** | Ambient + Placebo |  | 0.26±(0.02) |  | 0.62±(0.37) | 7.52±(2.52) | 2.96±(1.7) | 1.42±(0.37) | 6.36±(3.59) | 2.81±(2.57) |
|  | Ambient + Disease |  | 0.41±(0.01) |  | 0.73±(0.32) | 8.68±(1.63) | 5.16±(2.13) | 1.98±(0.74) | 12.86±(6.94) | 3.76±(3.65) |
|  | NH4 + Placebo | 6.56±(3.49) | 7.84±(3.18) | 7.52±(2.63) | 10.34±(6.15) | 22.07±(5.23) | 9.73±(4.26) | 3.25±(1.13) | 7.94±(3.6) | 5.05±(2.41) |
|  | NH4 + Disease | 7.51±(2.94) | 8.18±(3.17) | 8.47±(1.9) | 8.98±(3.16) | 21.87±(4.67) | 10.22±(4.82) | 3.92±(1.9) | 12.71±(4.11) | 6.61±(3.25) |
| **PO_4_**  **(μM)** | Ambient + Placebo |  |  |  |  | 0.4±(0.05) | 0±(0.66) | 0±(0.22) | 0.09±(1.98) | 0.01±(0.69) |
|  | Ambient + Disease |  |  |  |  | 0.37±(0.11) | 0±(0.38) | 0±(0.12) | 0.4±(1.7) | 0.04±(1.01) |
|  | NH4 + Placebo |  |  |  |  | 0.21±(0.12) | 0±(1.71) | 0±(0.41) | 0.19±(1.49) | 0.04±(0.94) |
|  | NH4 + Disease |  |  |  |  | 0.25±(0.14) | 0.03±(0.41) | 0±(0.3) | 0.2±(2.19) | 0.02±(1) |
