# Supplementary material for: Genotypes of Acropora cervicornis in Florida show resistance to either elevated nutrients or disease, but not both in combination
Source: PLoS One. 2025 Mar 26;20(3):e0320378. doi: 10.1371/journal.pone.0320378 (PMC11940558; doi:10.1371/journal.pone.0320378)
Supplement: S3 Table — (DOCX) [file pone.0320378.s004.docx]

**S3 Table. *Fv/Fm* model.** Type III analysis of variance table with Satterthwaite's method for the *Fv/Fm* linear mixed model

| **Factor** | **Sum Sq** | **Mean Sq** | **Num DF** | **Den DF** | **F value** | **Pr(>F)** |
| --- | --- | --- | --- | --- | --- | --- |
| Genotype | 0.187 | 0.021 | 9 | 227.051 | 30.082 | 3.459x10^-34^ |
| Nutrients | 0.006 | 0.006 | 1 | 226.968 | 9.0745 | 2.886x10^-03^ |
| Day | 0.502 | 0.063 | 8 | 1785.126 | 90.742 | 1.571x10^-126^ |
| Genotype x Day | 0.143 | 0.002 | 72 | 1784.899 | 2.875 | 6.188x10^-14^ |
| Nutrients x Day | 0.097 | 0.012 | 8 | 1784.996 | 17.468 | 2.903x10^-25^ |
