# Supplementary material for: Genotypes of Acropora cervicornis in Florida show resistance to either elevated nutrients or disease, but not both in combination
Source: PLoS One. 2025 Mar 26;20(3):e0320378. doi: 10.1371/journal.pone.0320378 (PMC11940558; doi:10.1371/journal.pone.0320378)
Supplement: S4 Table — (DOCX) [file pone.0320378.s005.docx]

**S4 Table. *Fv/Fm* pairwise comparisons among *A. cervicornis* genotypes.** Tukey HSD comparisons among the genotypes (time points combined). Alpha value = 0.05.

| **Genotype** | **emmean** | **SE** | **df** | **Lower CL** | **Upper CL** | **Tukey HSD Group** |
| --- | --- | --- | --- | --- | --- | --- |
| Kelsey-1 | 0.520 | 0.0041 | 227.278 | 0.512 | 0.528 | a |
| Acerv2 | 0.544 | 0.004 | 227.278 | 0.536 | 0.553 | b |
| Cooper-9 | 0.544 | 0.004 | 230.599 | 0.536 | 0.553 | b |
| Elkhorn | 0.559 | 0.004 | 238.125 | 0.5511 | 0.567 | bc |
| FM9 | 0.564 | 0.004 | 231.135 | 0.556 | 0.572 | c |
| FM19 | 0.570 | 0.004 | 225.841 | 0.562 | 0.578 | c |
| FM14 | 0.571 | 0.004 | 225.840 | 0.563 | 0.579 | c |
| FM6 | 0.572 | 0.004 | 225.840 | 0.563 | 0.579 | c |
| K2 | 0.577 | 0.004 | 234.700 | 0.569 | 0.586 | c |
| U44 | 0.606 | 0.004 | 225.840 | 0.597 | 0.614 | d |
