# Supplementary material for: Genotypes of Acropora cervicornis in Florida show resistance to either elevated nutrients or disease, but not both in combination
Source: PLoS One. 2025 Mar 26;20(3):e0320378. doi: 10.1371/journal.pone.0320378 (PMC11940558; doi:10.1371/journal.pone.0320378)
Supplement: S5 Table — (DOCX) [file pone.0320378.s006.docx]

**S5 Table. *Fv/Fm* Pairwise comparisons between the nutrient treatments.** Simple Tukey HSD comparisons among the treatments by sampling day. Alpha value = 0.05.

| **Nutrients** | **Day** | **emmean** | **SE** | **df** | **Lower CL** | **Upper CL** | **Group** |
| --- | --- | --- | --- | --- | --- | --- | --- |
| NH4 | -2 | 0.599 | 0.003 | 1081.729 | 0.593 | 0.605 | 1 |
| Ambient | -2 | 0.600 | 0.003 | 1097.126 | 0.595 | 0.606 | 1 |
| NH4 | 5 | 0.567 | 0.003 | 1081.730 | 0.561 | 0.572 | **1** |
| Ambient | 5 | 0.575 | 0.003 | 1102.492 | 0.570 | 0.581 | **2** |
| Ambient | 9 | 0.558 | 0.003 | 1099.985 | 0.552 | 0.564 | 1 |
| NH4 | 9 | 0.560 | 0.003 | 1081.730 | 0.554 | 0.565 | 1 |
| Ambient | 12 | 0.564 | 0.003 | 1107.725 | 0.558 | 0.570 | 1 |
| NH4 | 12 | 0.565 | 0.003 | 1089.466 | 0.560 | 0.571 | 1 |
| NH4 | 19 | 0.551 | 0.003 | 1089.466 | 0.545 | 0.557 | 1 |
| Ambient | 19 | 0.555 | 0.003 | 1107.725 | 0.550 | 0.561 | 1 |
| Ambient | 26 | 0.544 | 0.003 | 1107.735 | 0.539 | 0.550 | **1** |
| NH4 | 26 | 0.554 | 0.003 | 1097.714 | 0.548 | 0.560 | **2** |
| Ambient | 32 | 0.561 | 0.003 | 1107.746 | 0.556 | 0.567 | **1** |
| NH4 | 32 | 0.575 | 0.003 | 1106.866 | 0.569 | 0.581 | **2** |
| Ambient | 40 | 0.545 | 0.003 | 1107.759 | 0.539 | 0.551 | **1** |
| NH4 | 40 | 0.570 | 0.003 | 1117.018 | 0.564 | 0.576 | **2** |
| Ambient | 47 | 0.526 | 0.003 | 1117.280 | 0.520 | 0.532 | **1** |
| NH4 | 47 | 0.562 | 0.003 | 1207.046 | 0.556 | 0.568 | **2** |
