# Supplementary material for: Genotypes of Acropora cervicornis in Florida show resistance to either elevated nutrients or disease, but not both in combination
Source: PLoS One. 2025 Mar 26;20(3):e0320378. doi: 10.1371/journal.pone.0320378 (PMC11940558; doi:10.1371/journal.pone.0320378)
Supplement: S6 Table — (DOCX) [file pone.0320378.s007.docx]

**S6 Table. S/H cell ratio model.** Type III analysis of variance table with Satterthwaite's method for the S/H cell ratio linear mixed model.

| **Factor** | **Sum Sq** | **Mean Sq** | **Num DF** | **Den DF** | **F value** | **Pr(>F)** |
| --- | --- | --- | --- | --- | --- | --- |
| Genotype | 0.029 | 0.003 | 9 | 35.268 | 2.4352 | 0.028 |
| Nutrients | 0.001 | 0.001 | 1 | 3.989 | 0.033 | 0.865 |
| Nutrients x Genotype | 0.009 | 0.001 | 9 | 35.268 | 0.748 | 0.663 |
