# Supplementary material for: Genotypes of Acropora cervicornis in Florida show resistance to either elevated nutrients or disease, but not both in combination
Source: PLoS One. 2025 Mar 26;20(3):e0320378. doi: 10.1371/journal.pone.0320378 (PMC11940558; doi:10.1371/journal.pone.0320378)
Supplement: S7 Table — (DOCX) [file pone.0320378.s008.docx]

**S7 Table. S/H cell ratio pairwise comparisons among *A. cervicornis* genotypes.** Tukey HSD comparisons among the genotypes (time points combined). Alpha value = 0.05.

| **Genotype** | **emmean** | **SE** | **df** | **Lower CL** | **Upper CL** | **Tukey HSD Group** |
| --- | --- | --- | --- | --- | --- | --- |
| Kelsey-1 | 0.121 | 0.0165 | 31.5 | 0.0872 | 0.155 | a |
| Acerv2 | 0.180 | 0.0165 | 31.7 | 0.1465 | 0.214 | a |
| Cooper-9 | 0.197 | 0.0161 | 34.9 | 0.1644 | 0.230 | a |
| Elkhorn | 0.184 | 0.0165 | 31.6 | 0.1506 | 0.218 | a |
| FM9 | 0.152 | 0.0161 | 34.5 | 0.1194 | 0.185 | a |
| FM19 | 0.182 | 0.0157 | 36.3 | 0.1499 | 0.214 | a |
| FM14 | 0.190 | 0.0161 | 33.9 | 0.1574 | 0.223 | a |
| FM6 | 0.181 | 0.0161 | 34.8 | 0.1480 | 0.213 | a |
| K2 | 0.192 | 0.0165 | 31.1 | 0.1588 | 0.226 | a |
| U44 | 0.144 | 0.0188 | 30.9 | 0.1059 | 0.183 | a |
